# 11β-hydroxysteroid dehydrogenase type 1 has no effect on survival during experimental malaria but affects parasitemia in a parasite strain-specific manner

**DOI:** 10.1038/s41598-017-14288-x

**Published:** 2017-10-23

**Authors:** L. Vandermosten, C. De Geest, S. Knoops, G. Thijs, K. E. Chapman, K. De Bosscher, G. Opdenakker, P. E. Van den Steen

**Affiliations:** 1 Laboratory of Immunobiology, Department of Microbiology and Immunology, Rega Institute for Medical Research, KU Leuven – University of Leuven, Leuven, Belgium; 2 University/BHF Centre for Cardiovascular Science, The Queen’s Medical Research Institute, University of Edinburgh, Edinburgh, United Kingdom; 3 Receptor Research Laboratories, Nuclear Receptor Lab, VIB-UGent Center for Medical Biotechnology, Gent, Belgium

## Abstract

Malaria is a global disease associated with considerable mortality and morbidity. An appropriately balanced immune response is crucial in determining the outcome of malarial infection. The glucocorticoid (GC) metabolising enzyme, 11β-hydroxysteroid dehydrogenase-1 (11β-HSD1) converts intrinsically inert GCs into active GCs. 11β-HSD1 shapes endogenous GC action and is immunomodulatory. We investigated the role of 11β-HSD1 in two mouse models of malaria. 11β-HSD1 deficiency did not affect survival after malaria infection, but it increased disease severity and parasitemia in mice infected with *Plasmodium chabaudi* AS. In contrast, 11β-HSD1 deficiency rather decreased parasitemia in mice infected with the reticulocyte-restricted parasite *Plasmodium berghei* NK65 1556Cl1. Malaria-induced antibody production and pathology were unaltered by 11β-HSD1 deficiency though plasma levels of IL-4, IL-6 and TNF-α were slightly affected by 11β-HSD1 deficiency, dependent on the infecting parasite. These data suggest that 11β-HSD1 is not crucial for survival of experimental malaria, but alters its progression in a parasite strain-specific manner.

## Introduction

Malaria is a global health problem with an estimated 200 million clinical cases and 438,000 deaths in 2015^1^. Usually, malaria manifests as a mild febrile disease but complications can arise and include cerebral malaria (CM), severe malarial anaemia, placental malaria, hypoglycaemia and malaria-associated acute respiratory distress syndrome (MA-ARDS). Current antimalarial drugs effectively inhibit parasite growth but cannot resolve malaria complications, resulting in a mortality of about 15% for CM and up to 80% for MA-ARDS^2–4^. The immune response to malaria needs to be well balanced in order to control the infection without causing an overwhelming inflammation^5–7^. Inflammatory processes are thought to contribute significantly to the pathogenesis of specific malaria complications. For example, MA-ARDS is characterized by alveolar inflammation with infiltrating monocytes, macrophages, lymphocytes and neutrophils^3^. Anti-inflammatory molecules are essential to prevent immunopathology and include interleukin-10 (IL-10), transforming growth factor-β (TGF-β), heme oxygenase 1 (HO-1) and cytotoxic T-lymphocyte–associated antigen 4 (CTLA-4)^8–13^. In previous studies, an increase in blood cortisol levels was reported in *P*. *falciparum-* or *P*. *vivax*-infected patients, compared to healthy controls^14–22^. Cortisol levels decline during treatment as the clinical symptoms and parasitemia decrease^23^. One study reported that cortisol levels are higher in *P*. *falciparum-*infected patients with CM compared to uncomplicated malaria^14^. As glucocorticoids (GCs) have broad and potent immune-modulatory effects, they might be important in malaria to protect against excessive inflammation and its related complications. However, in two clinical trials conducted about 30 years ago, dexamethasone was tested in patients with CM without any benefit^24,25^. Nevertheless, and despite the established findings of increased endogenous GC levels in patients, surprisingly little is known about their importance and precise role during malaria infection.

Within cells, GC levels are regulated by 11β-hydroxysteroid dehydrogenase-1 and -2 (11β-HSD1 and 2) enzymes, which activate and inactivate endogenous GCs, respectively^26^. 11β-HSD1 amplifies intracellular GC levels (intracrine effects) and in addition contributes to ~30–40% of total active cortisol production in humans (endocrine effect) via the splanchnic bed which consists of the liver (highly expressing 11β-HSD1), gut and mesentery^26^. Knock-out of 11β-HSD1 abolishes GC regeneration while basal plasma corticosterone levels are maintained due to enhanced adrenal production^27^.

11β-HSD1 is widely expressed in immune cells and is regulated in a cell-specific manner, dependent on the cell activation state. During inflammation, when the hypothalamic-pituitary-adrenal (HPA) axis is activated, 11β-HSD1 is especially upregulated in activated immune cells, further amplifying endogenous GCs^26,28^. 11β-HSD1 expression is enhanced in dendritic cells (DCs) upon differentiation and innate immune induced maturation. This enhances the GC bioavailability and thus negatively regulates full DC differentiation, maturation, plasmacytoid DC survival and DC function^29,30^. 11β-HSD1 is important in restraining acute inflammation. The number of recruited inflammatory cells is increased in 11β-HSD1-deficient mouse models of inflammation in which neutrophils predominate in the inflammatory response^31^. Moreover, the switch from a pro-inflammatory, injury or tissue damaging (M1) response to a pro-resolution repair phase (M2) occurs earlier in 11β-HSD1 knock out mice^28^. Following LPS stimulation *in vitro*, macrophages are polarized to an M1 phenotype with high expression of 11β-HSD1^28^. 11β-HSD1-deficient mice are more susceptible to sublethal dose LPS endotoxemia as shown by an elevated weight loss and increased circulatory levels of pro-inflammatory tumor necrosis factor-α (TNF-α), IL-6, and IL-12p40. This was attributed to a macrophage hypersensitive state with increased signalling of nuclear factor-κB (NF-κB) and mitogen-activated protein kinase (MAPK)^32^. It was suggested that 11β-HSD1 alters the phenotypic differentiation rather than the glucocorticoid responsiveness of myeloid cells. In other models of acute inflammation, 11β-HSD1 deficiency caused an earlier onset of inflammation (K/BxN serum transfer model of inflammatory arthritis) or recruitment of more neutrophils and monocytes/macrophages (myocarditis), consistent with a role for 11β-HSD1 in restraining acute inflammation^33,34^. The effect of 11β-HSD1 is not always beneficial as its deficiency or inhibition is associated with better recovery of heart function after coronary artery ligation, probably because in the infarct zone, macrophages accumulate and polarise earlier to an M2 phenotype^34^. Furthermore, peripheral tissue regeneration of GCs by 11β-HSD1, rather than circulating corticosterone levels, mediates the Cushingoid effects of circulating GC excess^35^.

The objective of the current study was to investigate the role of peripheral reactivation of endogenous GCs by 11β-HSD1 in murine malarial disease. Therefore, we infected 11β-HSD1-deficient C57Bl/6 mice with two strains of murine *Plasmodium* parasites: *P*. *chabaudi* AS (*Pc*AS) and *Plasmodium berghei* NK65 (*Pb*NK65) 1556Cl1. Both these murine models are free of early lethality and thus serve well to observe even modest differences in disease course. *Pc*AS-infected C57Bl/6 mice are able to clear the parasite and survive the infection despite a transient phase of liver inflammation^36^. This model is frequently used to study immune mechanisms since a parasite-controlling immune response develops. Important differences exist between the red blood cell (RBC) subset preference of malaria parasites. Some parasites are able to infect normocytes (e.g. *P*. *falciparum* and *Pc*AS), while other parasites such as *P*. *vivax*, are restricted to reticulocytes^37^. To address this specificity, we also used the reticulocyte-restricted 1556Cl1 clone of *Pb*NK65 which can cause late-stage hyperparasitemia.

## Materials and Methods

### Mice

In all experiments, 8–10 week old male and female mice were used and each experimental and control group contained similar numbers of each sex. All animal experiments were approved by the Animal Ethics Committee from the KU Leuven (License LA1210186, Belgium) and conformed to the European Directive 2010/63/EU and the UK Animals (Scientific Procedures) Act 1986, as amended in 2012.

Mice homozygous for a null mutation in the gene encoding 11β-HSD1 (*Hsd11b1* ^*Del/Del*^) on a C57Bl/6 genetic background have been described^38^ and were generated by crossing mice homozygous for an allele of *Hsd11b1* in which exon 3 is flanked by *LoxP* sites^39^ with mice expressing *Cre* recombinase under the control of the *Hprt* promoter, expressed in the germ line^40^. To generate non-transgenic littermates as wild-type (WT) controls, C57Bl/6 mice were mated with *Hsd11b1* ^*Del/Del*^ mice to yield F1 heterozygotes. The latter were inter-crossed to obtain *Hsd11b1* ^*Del/Del*^ mice and matched WT controls in the F2 generation. Overall, *Hsd11b1* ^*Del/Del*^ mice were backcrossed to C57Bl/6 mice for more than 6 generations. Strain background strongly influences the course of malarial infection in mice. To characterize the percentage of C57Bl/6 background of WT and *Hsd11b1* ^*Del/Del*^ mice, background strain characterization was performed on genomic tail DNA from 3 *Hsd11b1* ^*Del/Del*^ mice and 3 of their WT littermates (Taconic, Rensselaer, NY, USA). A panel of 1449 SNP markers, covering all chromosomes, was compared to a C57Bl/6 J reference strain. This confirmed that *Hsd11b1* ^*Del/Del*^ mice showed 99.93–100% similarity to the C57Bl/6 reference genome. The characterization of all SNPs can be found as Supplementary Table S1.

### Infection of mice and clinical scoring

As described previously^41^, mice were housed in a conventional animal house and intraperitoneally injected with 10^4^ RBCs infected with *Pc*AS or *Pb*NK65 clone 1556Cl1 (a kind gift of Prof. C.J. Janse, Leiden University Medical Center, The Netherlands; corresponding to *Pb*NK65 NY strain^42^). Mice received high energy food *ad libitum* and drinking water was supplemented with para-amino benzoic acid (PABA; Sigma-Aldrich, Bornem, Belgium) to facilitate *in vivo* parasite growth.

Body weight, parasitemia, and clinical parameters including spontaneous activity (SA), limb grasping (LG), body tone (BT), trunk curl (TC), pilo-erection (PE), shivering (Sh), abnormal breathing (AB), dehydration (D), incontinence (I) and paralysis (P) were evaluated daily to calculate a clinical score of disease severity. A disease score was given of 0 (absent) or 1 (present) for TC, PE, Sh and AB and 0 (normal), 1 (intermediate) or 2 (most serious) for the other parameters. The total clinical score was calculated by the following formula: SA + LG + BT + TC + PE + Sh + AB + 3*(D + I + P). Peripheral parasitemia (percentage of RBCs that are infected) was determined by microscopic analysis of blood smears after Giemsa staining (VWR, Leuven, Belgium). Mice were euthanized when the humane endpoints were reached (clinical score of 15 or more) or at the indicated time points.

### Collection of tissues

Upon euthanasia, blood was collected by heart puncture. The left lung was pinched off and bronchoalveolar lavage fluid (BALF) was collected from the right lung as described previously^41^. Mice were systemically perfused (transcardial route) with PBS to remove circulating blood. Organs were removed, weighed and stored at −80 °C.

### Determination of glucose, protein and cytokine levels and assessment of liver pathology

Blood samples were centrifuged and plasma glucose concentrations were measured in duplicate or triplicate using a OneTouch Verio meter (LifeScan, Zurich, Switzerland). Plasma levels of interferon-γ (IFN-γ), TNF-α, IL-4, IL-5 and IL-6 were determined with a ProcartaPlex multiplex immunassay panel (EPX060-20831-901, Thermo Fisher Scientific Inc., Waltham, MA, USA) according to the manufacturer’s protocol. Liver function was assessed by measurement of plasma alanine aminotransferase (ALT) and aspartate transaminase (AST) using a reagent set according to the manufacturer’s protocols (Teco Diagnostics, California, USA). BALF samples were centrifuged and protein concentrations of the supernatants were determined by Bradford assay (Bio-Rad, Hercules, CA, USA).

### Preparation of crude parasite antigen

A crude parasite antigen was prepared from blood from 10 *Pc*AS-infected mice with a method modified from Elliot *et al*.^43^. The serum was removed from the blood by centrifugation (15 min. at 524 g) and the remaining pellet was washed three times with PBS (centrifugation for 10 min. at 3,000 g). The pellet was resuspended in PBS and cells were lysed with saponin (0.01%) for 20 min at 37 °C. The pellet from the lysate was washed with PBS (centrifugation for 20 min. at 20,000 g) until the supernatant was clear and colourless, resuspended in water and sonicated. The protein concentration was determined by Bradford analysis.

### Measurement of malaria-specific antibody levels in plasma

Circulating plasma levels of anti-parasitic IgG and IgM were determined by a direct ELISA with a method modified from Li *et al*.^44^ as reported previously^45^. 96 well NUNC immunoplates were coated overnight at 4 °C with the crude parasite antigen (5 µg/ml in 0.1 M NaHCO_3_, pH 9.6). After washing and blocking (in 1% BSA and 0.1% Tween 20 in PBS), serial dilutions of plasma samples and a laboratory standard were added to the wells and the plate was incubated for 1 h at 37 °C. The laboratory standard was prepared by pooling plasma samples from several mice, 21 days post-infection, and was given an arbitrary concentration of 90,000 laboratory standard units (LSU) per ml. For detection, 1/5,000 HRP-conjugated anti-mouse IgG or IgM (respectively, Fcγ or µ-chain specific, Jackson-Immunoresearch, West Grove, PA, USA) was added. The plate was incubated for 1 h at 37 °C, washed and HRP activity was visualized by adding TMB (0.55 µM, Sigma-Aldrich) and H_2_O_2_ (0.004% Merck, Darmstadt, Germany), in citrated acetate buffer (0.1 M) pH 4.3. The reaction was stopped with H_2_SO_4_ (1 M) and optical densities were determined at 450 nm on a microplate spectrophotometer (Power Wave XS, Biotek, Winooski, VT, USA).

### Statistical analysis

GraphPad Prism Software (GraphPad Software, San Diego, CA) was used for all statistical analyses. The non-parametric two-tailed Mann-Whitney U-test was used to calculate significance levels for the differences between groups. The non-parametric two-tailed Fisher exact test was used to compare proportions between two groups. GraphPad Prism software was also used to calculate the significance levels for the survival curves with the Log-rank (Mantel-Cox) Test. *p < 0.05, **p < 0.01, ***p < 0.001.

### Data availability

All data generated or analysed during this study are included in this published article and its Supplementary Information files.

## Results

### 11β-HSD1 deficiency does not affect survival upon *Pc*AS infection but changes parasitemia and disease severity

**Figure 1:**
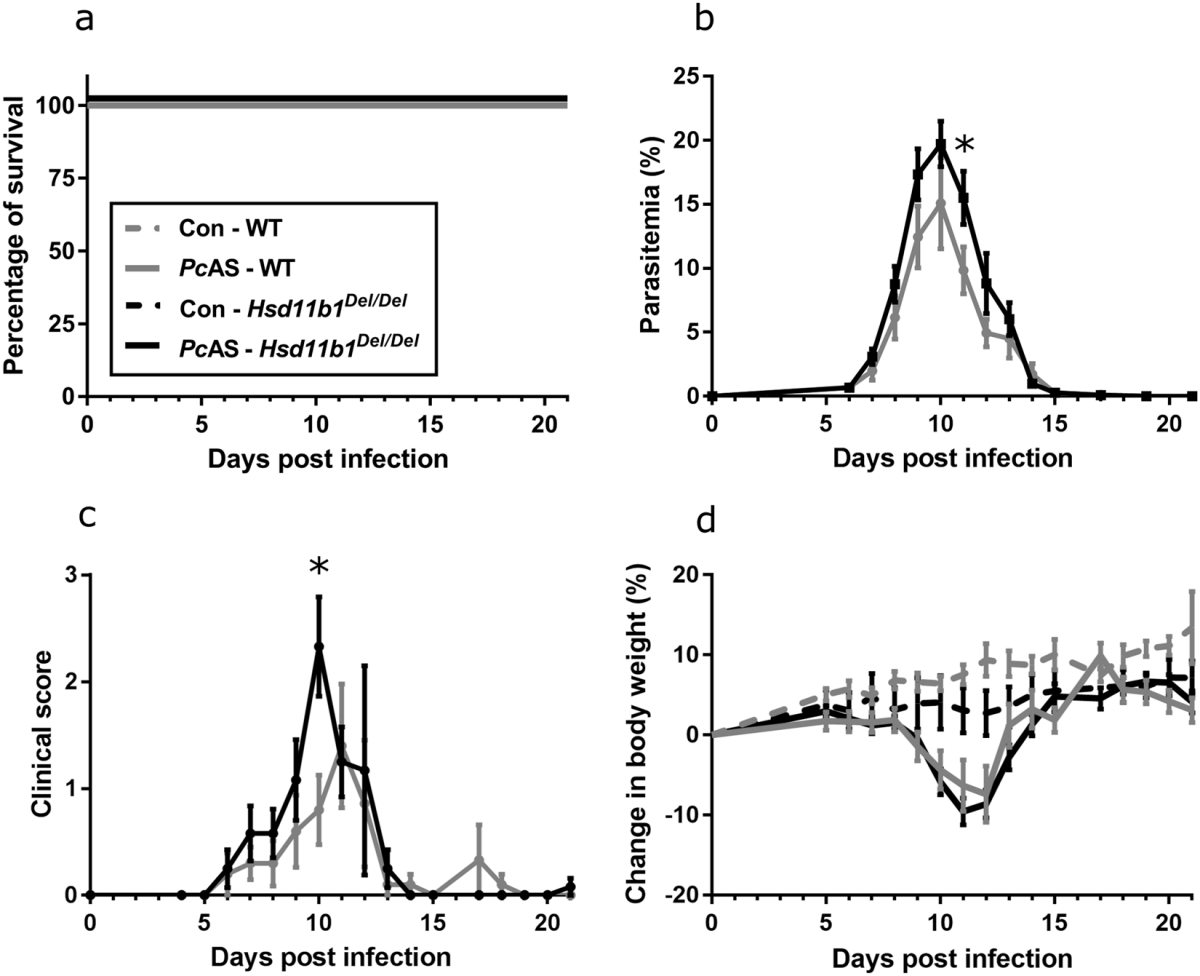
11β-HSD1 deficiency does not affect survival, but it increases parasitemia and clinical disease score in *Pc*AS-infected mice. *Hsd11b1* ^*Del/Del*^ and WT mice were infected with *Plasmodium chabaudi* AS (*Pc*AS). (**a**) The survival was monitored daily. The peripheral parasitemia (**b**), clinical score (**c**) and body weight (**d**) of the mice was determined at the indicated time points. Survival was analysed by Log-rank test. Data are means ± SEM and were analysed by Mann-Whitney U-test. Asterisks on top indicate a difference between the infected *Hsd11b1* ^*Del/Del*^ and WT mice. Data from 2 separate experiments. n = 10 (6 females (F) and 4 males (M)) for WT and n = 12 (6 F and 6 M) for *Hsd11b1* ^*Del/Del*^.

To determine whether peripheral reactivation of endogenous GCs has an effect on malarial disease, *Hsd11b1* ^*Del/Del*^ and WT mice were infected with the malarial parasite *Pc*AS. All *Pc*AS-infected *Hsd11b1* ^*Del/Del*^ and WT mice survived to the end of the experiment (Fig. 1a). The parasitemia showed a modest but significant (p = 0.04) increase at day 11 post-infection in *Hsd11b1* ^*Del/Del*^ mice compared to WT littermates (Fig. 1b). Also, the peak clinical score occurred one day earlier and was greater (p = 0.01) in *Hsd11b1* ^*Del/Del*^ mice compared to WT (Fig. 1c). Spontaneous activity, body tone and limb grasping determined the increase in clinical score, while the other, more severe used scoring parameters were rarely observed. No difference in body weight loss was observed between both genotypes (Fig. 1d).

Thus, 11β-HSD1 deficiency does not affect the survival upon infection with *Pc*AS, despite increased parasitemia and clinical score.

### 11β-HSD1 influences IL-4 levels but is not essential for the production of antimalarial antibodies or the prevention of malaria-associated pathology

**Figure 2:**
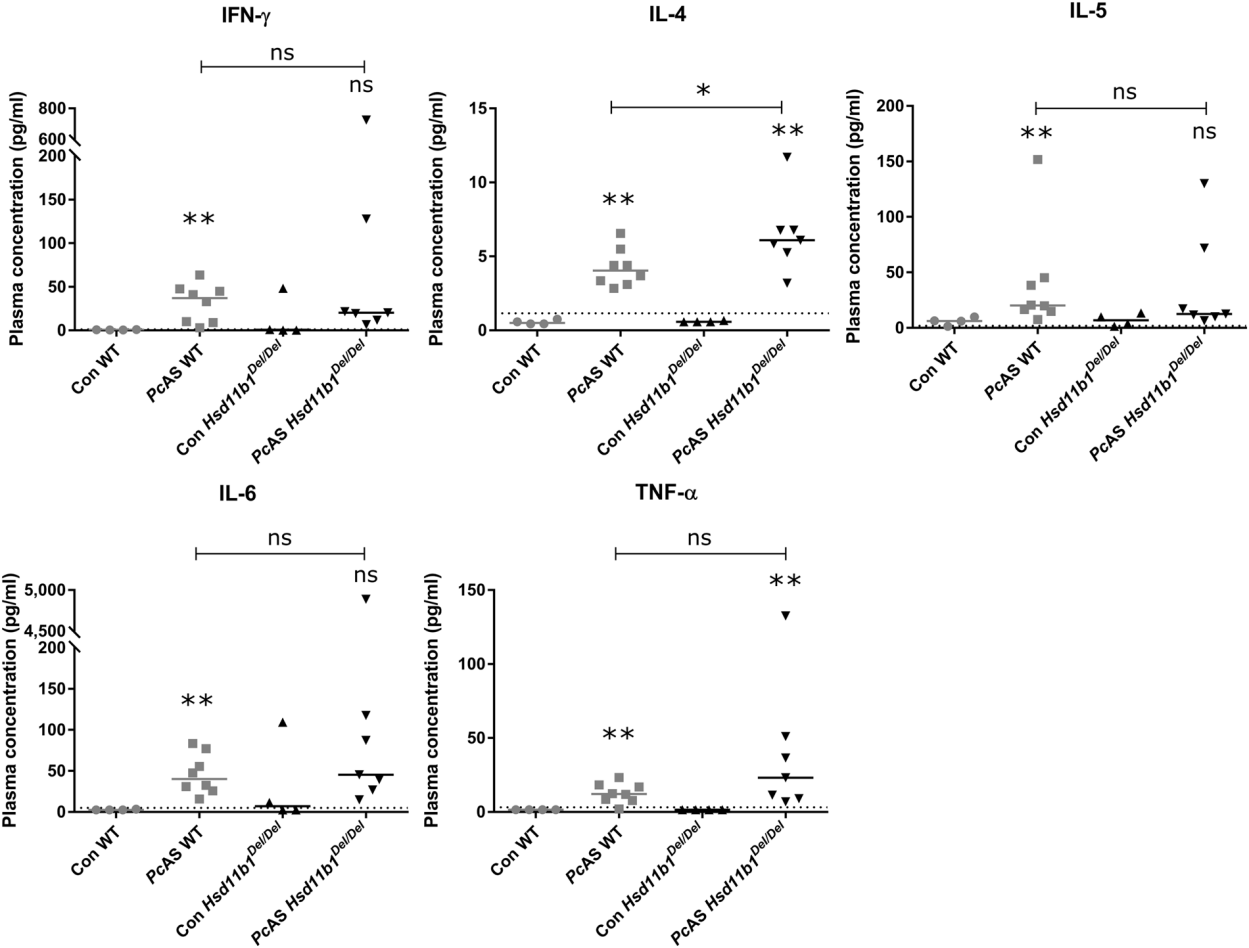
The effect of 11β-HSD1 on plasma levels of IFN-γ, IL-4, IL-5, IL-6 and TNF-α upon infection with *Pc*AS. *Hsd11b1* ^*Del/Del*^ and WT mice were infected with *Pc*AS. At the peak of infection, 10 days post-infection, plasma levels of IFN-γ, IL-4, IL-5, IL-6 and TNF-α were measured. The dotted line indicates the limit of detection and samples with a measurement below the detection limit, were given an arbitrary value of half of the detection limit. Each dot represents the result from an individual mouse. Horizontal lines in between data points represent group medians and analysis was by Mann-Whitney U-test. Asterisks above individual data sets indicate statistical differences compared to the uninfected control group. n = 8 (4 F and 4 M) for WT and n = 7 (4 F and 3 M) for *Hsd11b1* ^*Del/Del*^. ns, not significant.

To evaluate the elicited cytokine response, plasma levels of several cytokines were determined at peak parasitemia, 10 days post-infection (Fig. 2). Circulatory IFN-γ, IL-4, IL-5, IL-6 and TNF-α were increased in WT mice after infection with PcAS (respectively, p = 0.004, 0.004, 0.0081, 0.004 and 0.004). In *Hsd11b1* ^*Del/Del*^ mice, only IL-4 and TNF-α were significantly increased in response to the infection (respectively, p = 0.0061, 0.0061), and non-significant trends were noted for IFN-γ, IL-5 and IL-6 (respectively, p = 0.103, 0.109, 0.103). Notably, levels of IL-4 were increased to a greater extent in *Hsd11b1* ^*Del/Del*^ mice than in WT (p = 0.037) while IFN-γ, IL-5, IL-6 and TNF-α plasma concentrations did not differ between genotypes (p = 0.96, 0.40, 0.54, 0.28, respectively).

**Figure 3:**
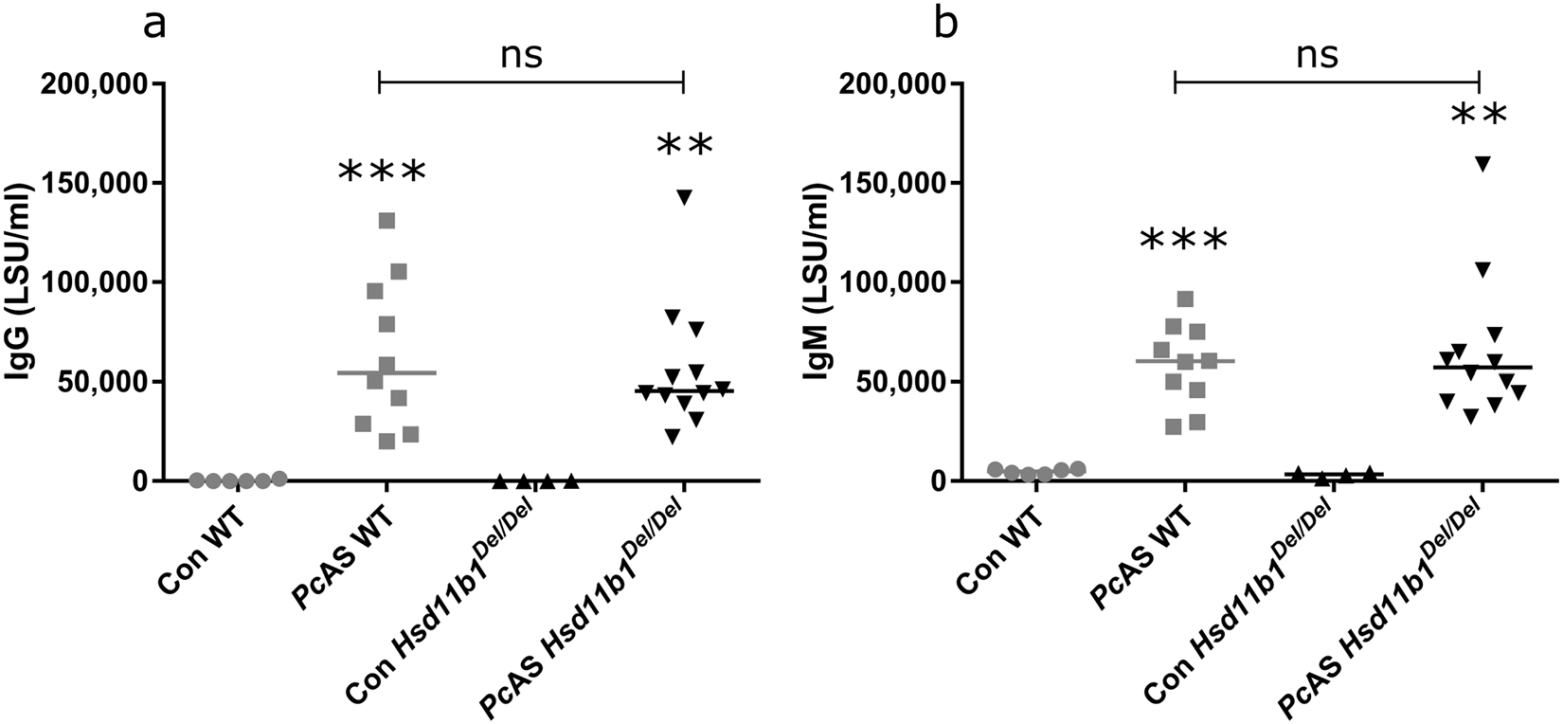
11β-HSD1 has no effect on the production of malaria-specific IgG and IgM. *Hsd11b1* ^*Del/Del*^ and WT mice were infected with *Pc*AS. Malaria-specific antibody levels in plasma were analysed by ELISA at 21 days post-infection. Each dot represents the result from an individual mouse. Horizontal lines in between data points represent group medians and analysis was by Mann-Whitney U-test. Asterisks above individual data sets indicate statistical differences compared to the uninfected control group. Data from 2 separate experiments. n = 10 (6 F and 4 M) for WT and n = 12 (6 F and 6 M) for *Hsd11b1* ^*Del/Del*^. ns, not significant.

Three weeks after infection with *Pc*AS, anti-parasitic IgG and IgM were increased in WT and *Hsd11b1* ^*Del/Del*^ mice (respectively p = 0.0002 and p = 0.0011 Fig. 3a; p = 0.0002 and p = 0.0011 Fig. 3b). However, there were no differences in antibody levels between *Pc*AS-infected *Hsd11b1* ^*Del/Del*^ and WT mice (p = 0.82 Fig. 3a and p = 0.97 Fig. 3b). This indicates that 11β-HSD1-deficiency has no effect on the humoral immune response against *Pc*AS parasites.

**Figure 4:**
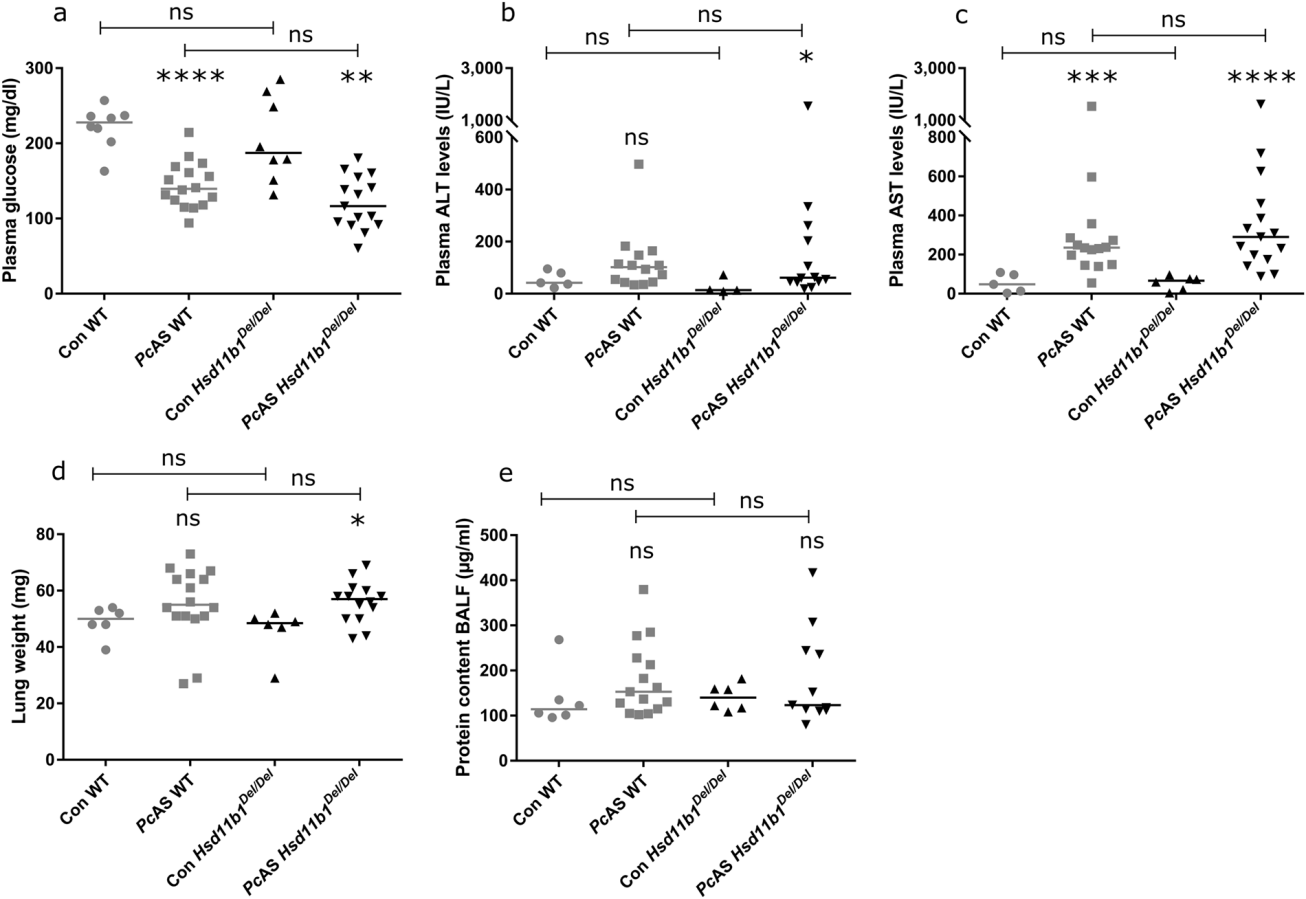
11β-HSD1 does not change plasma glucose, ALT or AST levels and does not affect lung pathology upon infection with *Pc*AS. *Hsd11b1* ^*Del/Del*^ and WT mice were infected with *Pc*AS. At the peak of infection, 10 days post-infection, plasma glucose levels, alanine aminotransferase (ALT) and aspartate transaminase (AST) levels were measured. Also, the weight of the left lung and the protein content in the bronchoalveolar lavage fluid (BALF) were determined. Each dot represents the result from an individual mouse. Horizontal lines in between data points represent group medians and analysis was by Mann-Whitney U-test. Asterisks above individual data sets indicate statistical differences compared to the uninfected control group. Data from 2 separate experiments. n = 16 (8 F and 8 M) for WT and n = 15 (8 F and 7 M) for *Hsd11b1* ^*Del/Del*^. ns, not significant.

Plasma glucose levels were measured, since GCs and 11β-HSD1 have metabolic effects besides their immune-modulatory properties and malaria-infection is known to affect glycaemia^26,46,47^. However, at peak parasitemia, 10 days post-infection, a similar (p = 0.088) decrease in plasma glucose levels was observed in *Pc*AS-infected *Hsd11b1* ^*Del/Del*^ and WT mice (p = 0.0014 and <0.0001, respectively), compared to uninfected mice (Fig. 4a).

Liver damage and lung inflammation may occur following malarial infection^36,41^. As an indicator of liver damage, plasma levels of ALT and AST were measured (Fig. 4b,c). At peak parasitemia, ALT levels were significantly increased in *Hsd11b1* ^*Del/Del*^ mice compared to uninfected controls (p = 0.035) and a similar trend was observed in WT mice (p = 0.11). AST levels were elevated in both *Hsd11b1* ^*Del/Del*^ (p < 0.0001) and WT mice (p = 0.0004) upon infection. However, neither ALT nor AST were significantly different between infected *Hsd11b1* ^*Del/Del*^ and WT mice (p = 0.98 and p = 0.57, respectively).

Lung inflammation was evaluated by measuring the lung weight and by determining the protein content in the BALF at peak parasitemia (Fig. 4d,e). Compared to uninfected controls, *Hsd11b1* ^*Del/Del*^ mice showed a modest but significant increase in lung weight (p = 0.015), while infected WT mice only showed a trend towards heavier lungs (p = 0.093). However, a more accurate assessment of lung pathology by protein content in the BALF did not indicate a significant alteration upon infection with *Pc*AS (p = 0.13 for WT and p = 0.88 for *Hsd11b1* ^*Del/Del*^). Moreover, these indicators of lung pathology did not differ between *Hsd11b1* ^*Del/Del*^ and WT mice (p = 0.80 for lung weight and p = 0.80 for BALF).

Overall, these data show that 11β-HSD1 deficiency increases IL-4 levels in response to *Pc*AS infection, but does not influence malaria-associated antibody production or pathology.

### Effect of 11β-HSD1 deficiency on infection with the reticulocyte-restricted *Pb*NK65 1556Cl1

*Pc*AS infects normocytes as well as reticulocytes. To investigate whether 11β-HSD1 deficiency also alters parasitemia and clinical score with a reticulocyte-restricted malarial strain, *Hsd11b1* ^*Del/Del*^ mice and their WT littermates were infected with *Pb*NK65 1556Cl1.

**Figure 5:**
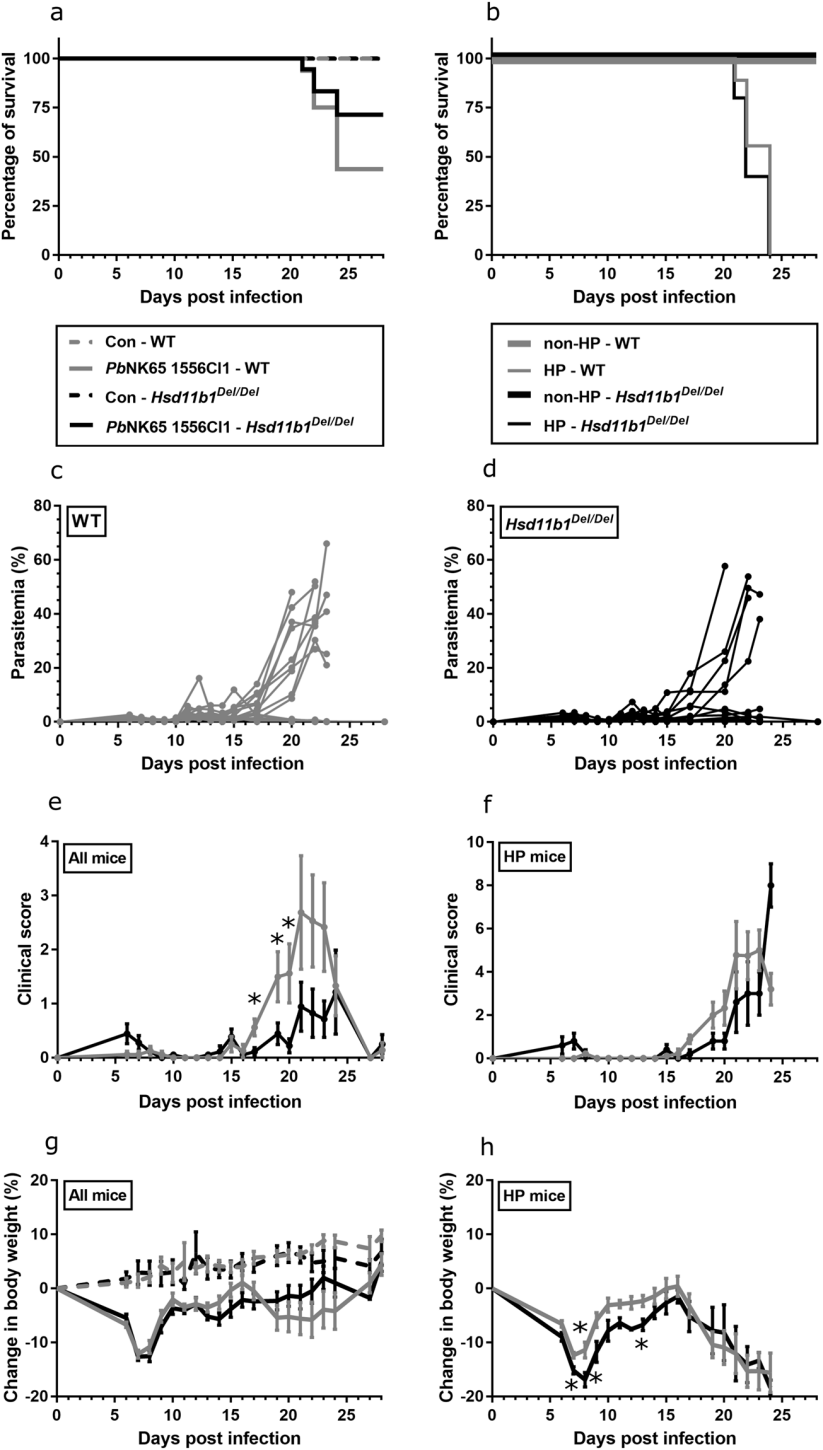
Effect of 11β-HSD1 on the disease and parasitemia course in *Pb*NK65 1556Cl1-infected mice. *Hsd11b1* ^*Del/Del*^ and WT mice were infected with *Plasmodium berghei* NK65 (*Pb*NK65) clone 1556Cl1 and a differentiation was made between hyperparasitemic (HP) and non-hyperparasitemic (non-HP) mice. For the clinical score and body weight of non-HP mice, see supplementary Fig. S1. Survival (**a**,**b**), individual peripheral parasitemia (**c**,**d**), clinical score (**e**,**f**) and body weight (**g**,**h**) of the mice is shown. Survival was analysed by Log-rank test. Data are means ± SEM and were analysed by Mann-Whitney U-test. Asterisks on top indicate a difference between the infected *Hsd11b1* ^*Del/Del*^ and WT mice. Data from 2 separate experiments. n = 16 (7 F and 9 M) for WT and n = 18 (9 F and 9 M) for *Hsd11b1* ^*Del/Del*^. n = 9 (3 F and 6 M) for HP WT and n = 5 (2 F and 3 M) for HP *Hsd11b1* ^*Del/Del*^.

A proportion of the infected mice developed lethal hyperparsitemia, whereas in the other mice, parasitemia remained below 6% during the observed course of the experiment. There was a trend towards a higher survival in *Pb*NK65 1556Cl1-infected *Hsd11b1* ^*Del/Del*^ compared to WT mice (Log-rank test p = 0.14; Fig. 5a), in line with a trend for fewer *Hsd11b1* ^*Del/Del*^ mice developing lethal hyperparasitemia compared to WT mice (27.78% *versus* 56.25%; Fisher exact test p = 0.16) (Fig. 5c,d). The trend towards a reduction in lethal hyperparasitemia in *Hsd11b1* ^*Del/Del*^ mice compared to WT was paralleled by a lower group mean clinical score in the third week of infection (Fig. 5e; p = 0.020, 0.037, 0.047 for respectively d17, 19, 20) without an effect on body weight (Fig. 5g). Hyperparasitemic (HP) *Hsd11b1* ^*Del/Del*^ and WT mice died at a similar time point, 21 to 24 days after infection (Fig. 5b). When the analysis was restricted to HP or non-HP mice only, no difference in the clinical score was found between genotypes (Fig. 5f and Supplementary Fig. S1). Clinical symptoms were either absent or very mild in non-HP mice. Interestingly, of the mice that developed hyperparasitemia, *Hsd11b1* ^*Del/Del*^ mice showed a greater body weight loss compared to WT within the first two weeks of infection, when parasitemia levels were still low (Fig. 5h; p = 0.013, 0.017, 0.042, 0.020 respectively, for d7–9 and d13). This difference in body weight loss was not observed in the non-HP mice (Supplementary Fig. S1).

**Figure 6:**
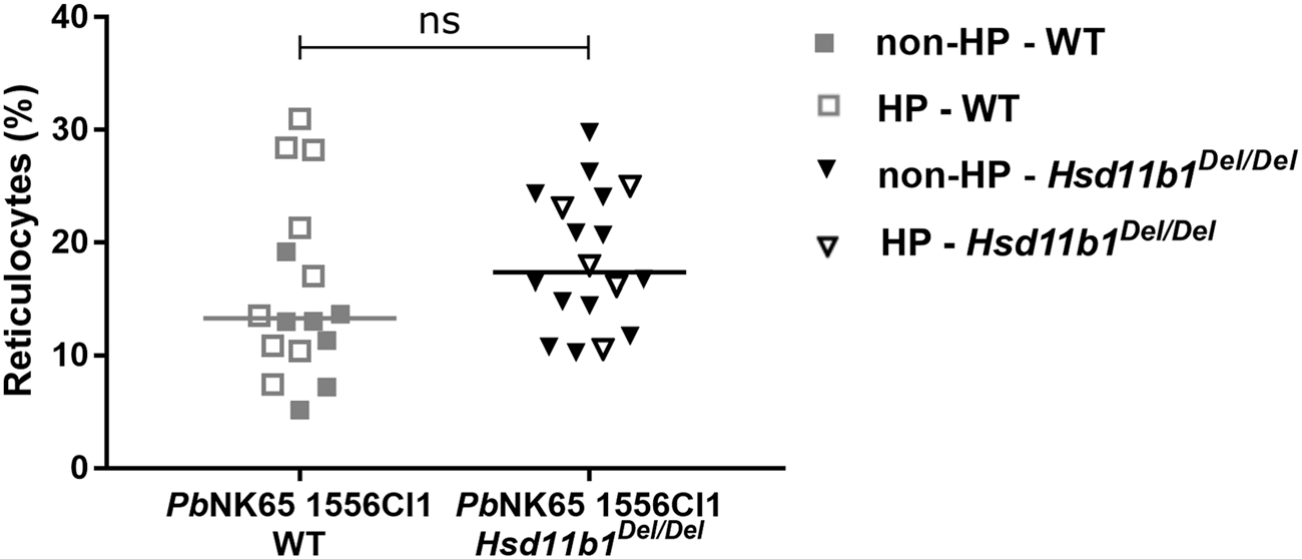
Reticulocyte presence is not affected by 11β-HSD1. *Hsd11b1* ^*Del/Del*^ and WT mice were infected with *Pb*NK65 1556Cl1. The percentage of reticulocytes amongst the RBCs was analysed by microscopical examination of blood smears at 17 days post-infection. Each dot represents the result from an individual mouse. Horizontal lines in between data points represent group medians and analysis was by Mann-Whitney U-test. Data from 2 separate experiments. n = 16 (7 F and 9 M) for WT and n = 18 (9 F and 9 M) for *Hsd11b1* ^*Del/Del*^. HP, hyperparasitemic; non-HP, non-hyperparasitemic; ns, not significant.

There was no significant difference in reticulocytosis between *Hsd11b1* ^*Del/Del*^ and WT mice at 17 days after infection (p = 0.20; Fig. 6). Also, the abundant reticulocytosis (16% in infected *versus* ≤3% in non-infected mice) occurred both in HP and non-HP mice (Fig. 6). Only in non-HP mice, a lower reticulocytosis was noted in WT mice compared to *Hsd11b1* ^*Del/Del*^ mice (p = 0.017; Supplementary Fig. S2).

The levels of IFN-γ, IL-4, IL-5, IL-6 and TNF-α were measured in the plasma of HP and non-HP *Pb*NK65 1556Cl1-infected *Hsd11b1* ^*Del/Del*^ and WT mice at 21 to 24 days (HP) or 28 days (non-HP) after infection (Supplementary Fig. S3). Compared to uninfected controls, cytokine levels of non-HP mice remained at baseline, while in HP mice, the levels were increased upon infection. In the HP mice, a higher increase of IL-6 levels (p = 0.030) and a trend towards a higher increase of TNF-α levels (p = 0.052) were observed in *Hsd11b1* ^*Del/Del*^ versus WT mice.

Taken together, we have shown that 11β-HSD1 deficiency resulted in a trend towards less lethal hyperparasitemia with the reticulocyte-restricted parasite strain *Pb*NK65 1556Cl1. This was paralleled by increased plasma levels of IL-6.

## Discussion

To determine whether peripheral reactivation of endogenous GCs has an effect on malarial disease, we studied the phenotype of *Hsd11b1* ^*Del/Del*^ mice. 11β-HSD1 deficiency altered parasitemia and clinical disease score in mice infected with *Pc*AS or *Pb*NK65 1556Cl1. *Pc*AS-infected *Hsd11b1* ^*Del/Del*^ mice showed increased parasitemia and clinical score, whereas a trend was observed for fewer *Pb*NK65 1556Cl1-infected *Hsd11b1* ^*Del/Del*^ mice to develop lethal hyperparasitemia. Thus, 11β-HSD1 clearly influences parasitemia in a parasite strain-dependent manner.

*Pc*AS has been frequently used to study immune mechanisms, as resistant mouse strains such as C57Bl/6 mice develop an immune response that controls the parasite. To control the initial replication of the parasite, innate immune rather than antibody-mediated mechanisms are essential^48^. Both DCs and macrophages are central in the innate immune response to the parasite. DCs endocytose the parasite, produce IL-12 and present antigens to CD4^+^ T cells^48^. Macrophages are able to phagocytose infected RBCs via CD36 and they sense parasite-associated components which trigger cytokine production^49,50^. 11β-HSD1 affects macrophages and DCs. The enzyme inhibits full DC differentiation, maturation, plasmacytoid DC survival and DC function^29,30^. Expression of 11β-HSD1 is induced upon differentiation of monocytes into macrophages and even more in M1 polarised macrophages^28^. McSweeney *et al*. showed that 11β-HSD1 deficient mice switch earlier to an M2 phenotype after myocardial infarction^34^. Here, we observed mildly increased levels of IL-4 in *Hsd11b1* ^*Del/Del*^ mice compared to WT mice upon *Pc*AS infection. IL-4 can stimulate M2 macrophage activation which may mirror a Th2 polarization^51^. Th1/M1 rather than Th2/M2 type reactions are crucial for an efficient anti-malarial immunity, especially in the early stage^6^. IL-4 slightly delays parasite clearance in P. yoelii 17XNL-infected mice, although it can also suppress recrudescences upon *Pc*AS infection^52,53^. The increase in IL-4 production in 11β-HSD1 deficient mice might thus indicate a greater or earlier polarisation to M2 macrophages, resulting in a less efficient parasite clearance and the observed increase in parasitemia. The generation of cell-specific 11β-HSD1 knockout mice may provide useful tools to identify the importance of 11β-HSD1 in macrophages or DCs during malaria infection. After the initial phase of innate immunity, the adaptive immunity plays an important role in clearing the infection^6^. Anti-parasitic antibody levels did not differ between WT and *Hsd11b1* ^*Del/Del*^ mice. Therefore, the effect of 11β-HSD1 on PcAS parasitemia was not attributed to a change in parasite-specific antibody production.

The parasitemia course and the effect of 11β-HSD1 on the parasitemia differs between *Pb*NK65 1556Cl1 and *Pc*AS-infected mice. A difference in infected RBC type, in provoked immune response or both might be the cause of the opposite effects in the two models. Regarding the infected RBC type, we showed in preliminary experiments that *Pb*NK65 1556Cl1 parasites are restricted to reticulocytes and do not infect normocytes, in accordance to the findings by Deharo *et al*.^54^. These infections thus depend on erythropoiesis, as parasitemia is limited by the number of available reticulocytes. Hyperparasitemia can therefore only occur when high reticulocytosis is induced due to anemia. GCs may increase erythropoiesis during malaria infection by stimulating self-renewal of an early erythroid progenitor^55^. However, deficiency in 11β-HSD1 did not impede the erythropoiesis upon infection, since similar numbers of reticulocytes were found in both genotypes. Here, a proportion of *Pb*NK65 1556Cl1-infected mice developed hyperparasitemia at a late stage of infection, together with a considerable reticulocytosis. The reasons why some mice do not develop hyperparasitemia are not related to reticulocytosis, and could therefore be the consequence of the immune response. The role of both innate and adaptive immunity on parasite control is complex and has been widely investigated^6^. In HP *Pb*NK65 1556Cl1-infected mice, IL-6 was significantly increased in the absence of 11β-HSD1, and a trend towards increased TNF-α was noted. This effect might reflect a decrease in anti-inflammatory GC activity. Previous work has established that in LPS endotoxemia, 11β-HSD1 deficiency is associated with higher levels of TNF-α and IL-6^32^. However, in contrast to *Pc*AS-infected mice, no difference in IL-4 response was seen in *Pb*NK65 1556Cl1-infected mice. This indicates that the effect of 11β HSD1 on the cytokine response depends on the parasite strain.

Despite the shift in parasitemia, 11β-HSD1 did not affect survival in the studied murine malaria models. Also, the malaria-associated pathology was not altered after infection with *Pc*AS. This is in contrast to LPS-induced inflammation which is aggravated in the absence of 11β-HSD1^32^. Furthermore, GCs themselves are essential to survive LPS-mediated endotoxemia or other infections, for example with murine cytomegalovirus or with bacteria that produce toxins such as *C*. *difficile* toxin A, Shiga toxin 2 or superantigen SEB^56–58^. Also, infections can trigger an Addisonian crisis in patients with adrenal insufficiency^59^. In this context, it should be noted that although 11β-HSD1 is essential for intracellular regeneration of GCs, 11β-HSD1 deficiency or inhibition is associated with increased adrenal production of GCs to maintain normal circulating levels^27,28^. Thus, we have shown that intracellular regeneration of GCs can modulate cytokine responses and the course of disease during malaria infection.

Blood glucose levels were decreased upon malaria infection to a similar extent in WT and *Hsd11b1* ^*Del/Del*^ mice, consistent with findings that 11β-HSD1 deficiency does not induce fasting hypoglycemia^60^. This indicates that glucose counter-regulatory responses are not affected by 11β-HSD1 deficiency despite the reduced induction of glucose-6-phosphatase and phosphoenolpyruvate carboxykinase activity upon starvation^26,60^. In contrast, in the case of hyperglycemia in obese or diabetic mice, 11β-HSD1 deficiency or inhibition of 11β-HSD1 results in lower fasting glucose levels, attributed to decreased hepatic gluconeogenesis and increased insulin sensitivity^26^.

Overall, this study indicates that 11β-HSD1 is not crucial for survival but alters parasitemia and cytokine response during malaria in a *Plasmodium* strain-specific manner. A major difference between the murine parasites, used here, is their RBC subset preference, with *Pb*NK65 being restricted to reticulocytes and *Pc*AS infecting normocytes. From this perspective, it may be hypothesized that 11β-HSD1 might also increase hyperparasitemia in patients with (reticulocyte restricted) *P*. *vivax* malaria, whereas it might limit parasitemia in *P*. *falciparum*-infected patients. In the future, it will be of interest to analyse *HSD11B1* genotype differences (SNPs) in malaria patients.

## Electronic supplementary material

Supplementary information

Supplementary Table S1

## References

[1] World Health Organisation. *World Malaria Report*. (2016).

[2] White NJ (2008). Qinghaosu (artemisinin): the price of success. Science.

[3] Van den Steen PE (2013). Pathogenesis of malaria-associated acute respiratory distress syndrome. Trends Parasitol..

[4] Taylor WRJ, Hanson J, Turner GDH, White NJ, Dondorp AM (2012). Respiratory manifestations of malaria. Chest.

[5] Riley EM, Stewart VA (2013). Immune mechanisms in malaria: new insights in vaccine development. Nat. Med..

[6] Deroost K, Pham T-T, Opdenakker G, Van den Steen PE (2016). The immunological balance between host and parasite in malaria. FEMS Microbiol. Rev..

[7] Storm J, Craig AG (2014). Pathogenesis of cerebral malaria–inflammation and cytoadherence. Front. Cell. Infect. Microbiol..

[8] Li C, Corraliza I, Langhorne J (1999). A defect in interleukin-10 leads to enhanced malarial disease in Plasmodium chabaudi chabaudi infection in mice. Infect. Immun..

[9] Sanni LA, Jarra W, Li C, Langhorne J (2004). Cerebral edema and cerebral hemorrhages in interleukin-10-deficient mice infected with Plasmodium chabaudi. Infect. Immun..

[10] Omer FM, Riley EM (1998). Transforming growth factor beta production is inversely correlated with severity of murine malaria infection. J. Exp. Med..

[11] Pamplona A (2007). Heme oxygenase-1 and carbon monoxide suppress the pathogenesis of experimental cerebral malaria. Nat. Med..

[12] Seixas E (2009). Heme oxygenase-1 affords protection against noncerebral forms of severe malaria. Proc. Natl. Acad. Sci. USA.

[13] Hafalla JCR (2012). The CTLA-4 and PD-1/PD-L1 inhibitory pathways independently regulate host resistance to Plasmodium-induced acute immune pathology. PLoS Pathog..

[14] Blümer RME (2005). Adiponectin and glucose production in patients infected with Plasmodium falciparum. Metabolism.

[15] Davis TME (1997). The hypothalamic-pituitary-adrenocortical axis in severe falciparum malaria: effects of cytokines. J. Clin. Endocrinol. Metab..

[16] Dekker E (1997). Glucose production and gluconeogenesis in adults with uncomplicated falciparum malaria. Am. J. Physiol..

[17] Enwonwu CO (1999). Hyperphenylalaninaemia in children with falciparum malaria. QJM.

[18] Muehlenbein MP, Alger J, Cogswell F, James M, Krogstad D (2005). The reproductive endocrine response to Plasmodium vivax infection in Hondurans. Am. J. Trop. Med. Hyg..

[19] Shwe T (1998). Serum cortisol levels in patients with uncomplicated and cerebral malaria. Southeast Asian J. Trop. Med. Public Health.

[20] van Thien H (2004). FFAs are not involved in regulation of gluconeogenesis and glycogenolysis in adults with uncomplicated P. falciparum malaria. Am. J. Physiol. Endocrinol. Metab..

[21] van Thien H (2001). Glucose production and gluconeogenesis in adults with cerebral malaria. QJM.

[22] Wilson M (2001). Pituitary-adrenal function in uncomplicated falciparum malaria. Southeast Asian J. Trop. Med. Public Health.

[23] Libonati RMF, de Mendonça BB, Maués JA, Quaresma JAS, de Souza JM (2006). Some aspects of the behavior of the hypothalamus-pituitary-adrenal axis in patients with uncomplicated Plasmodium falciparum malaria: Cortisol and dehydroepiandrosterone levels. Acta Trop..

[24] Warrell DA (1982). Dexamethasone proves deleterious in cerebral malaria. A double-blind trial in 100 comatose patients. N. Engl. J. Med..

[25] Hoffman SL (1988). High-dose dexamethasone in quinine-treated patients with cerebral malaria: a double-blind, placebo-controlled trial. J. Infect. Dis..

[26] Chapman K, Holmes M, Seckl J (2013). 11-Hydroxysteroid dehydrogenases: intracellular gate-keepers of tissue glucocorticoid action. Physiol. Rev..

[27] Carter RN (2009). Hypothalamic-pituitary-adrenal axis abnormalities in response to deletion of 11β-HSD1 is strain-dependent. J. Neuroendocrinol..

[28] Chapman KE (2013). Changing glucocorticoid action: 11β-hydroxysteroid dehydrogenase type 1 in acute and chronic inflammation. J. Steroid Biochem. Mol. Biol..

[29] Freeman L (2005). Expression of 11beta-hydroxysteroid dehydrogenase type 1 permits regulation of glucocorticoid bioavailability by human dendritic cells. Blood.

[30] Soulier A (2013). Cell-intrinsic regulation of murine dendritic cell function and survival by prereceptor amplification of glucocorticoid. Blood.

[31] Coutinho AE (2016). 11β-Hydroxysteroid dehydrogenase type 1 is expressed in neutrophils and restrains an inflammatory response in male mice. Endocrinology.

[32] Zhang TY, Daynes RA (2007). Macrophages from 11beta-hydroxysteroid dehydrogenase type 1-deficient mice exhibit an increased sensitivity to lipopolysaccharide stimulation due to TGF-beta-mediated up-regulation of SHIP1 expression. J. Immunol..

[33] Coutinho AE (2012). 11β-Hydroxysteroid dehydrogenase type 1, but not type 2, deficiency worsens acute inflammation and experimental arthritis in mice. Endocrinology.

[34] McSweeney SJ (2010). Improved heart function follows enhanced inflammatory cell recruitment and angiogenesis in 11betaHSD1-deficient mice post-MI. Cardiovasc. Res..

[35] Morgan Sa (2014). 11β-HSD1 is the major regulator of the tissue-specific effects of circulating glucocorticoid excess. Proc. Natl. Acad. Sci. USA.

[36] Deroost K (2014). Hemozoin induces hepatic inflammation in mice and is differentially associated with liver pathology depending on the Plasmodium strain. PLoS One.

[37] Malleret B (2015). Plasmodium vivax: restricted tropism and rapid remodeling of CD71-positive reticulocytes. Blood.

[38] Verma, M. *et al*. 11β-hydroxysteroid dehydrogenase-1 deficiency alters brain energy metabolism in acute systemic inflammation. Submitted, (2017). 10.1016/j.bbi.2017.11.015 PMC5871395 29162555

[39] White CI (2016). Cardiomyocyte and vascular smooth muscle-independent 11β-hydroxysteroid dehydrogenase 1 amplifies infarct expansion, hypertrophy, and the development of heart failure after myocardial infarction in male mice. Endocrinology.

[40] Tang S-HE, Silva FJ, Tsark WMK, Mann JR (2002). A Cre/loxP-deleter transgenic line in mouse strain 129S1/SvIm. J. Genesis.

[41] Van den Steen PE (2010). Immunopathology and dexamethasone therapy in a new model for malaria-associated acute respiratory distress syndrome. Am. J. Respir. Crit. Care Med..

[42] Otto TD (2014). A comprehensive evaluation of rodent malaria parasite genomes and gene expression. BMC Biol..

[43] Elliott SR, Kuns RD, Good MF (2005). Heterologous immunity in the absence of variant-specific antibodies after exposure to subpatent infection with blood-stage malaria. Infect. Immun..

[44] Li C, Langhorne J (2000). Tumor necrosis factor alpha p55 receptor is important for development of memory responses to blood-stage malaria infection. Infect. Immun..

[45] Geurts N (2011). Insufficiently defined genetic background confounds phenotypes in transgenic studies as exemplified by malaria infection in Tlr9 knockout mice. PLoS One.

[46] van Thien H, Kager PA, Sauerwein HP (2006). Hypoglycemia in falciparum malaria: is fasting an unrecognized and insufficiently emphasized risk factor?. Trends Parasitol..

[47] Phillips RE, Looareesuwan S, Molyneux ME, Hatz C, Warrell DA (1993). Hypoglycaemia and counterregulatory hormone responses in severe falciparum malaria: treatment with Sandostatin. Q. J. Med..

[48] Stevenson MM, Riley EM (2004). Innate immunity to malaria. Nat. Rev. Immunol..

[49] McGilvray ID, Serghides L, Kapus A, Rotstein OD, Kain KC (2000). Nonopsonic monocyte/macrophage phagocytosis of Plasmodium falciparum-parasitized erythrocytes: a role for CD36 in malarial clearance. Blood.

[50] Chua CLL, Brown G, Hamilton JA, Rogerson S, Boeuf P (2013). Monocytes and macrophages in malaria: Protection or pathology?. Trends Parasitol..

[51] Sica A, Mantovani A (2012). Macrophage plasticity and polarization: *in vivo* veritas. J. Clin. Invest..

[52] van der Heyde HC, Pepper B, Batchelder J, Cigel F, Weidanz WP (1997). The time course of selected malarial infections in cytokine-deficient mice. Exp. Parasitol..

[53] von der Weid T, Kopf M, Köhler G, Langhorne J (1994). The immune response to Plasmodium chabaudi malaria in interleukin-4-deficient mice. Eur. J. Immunol..

[54] Deharo E, Coquelin F, Chabaud AG, Landau I (1996). The erythrocytic schizogony of two synchronized strains of plasmodium berghei, NK65 and ANKA, in normocytes and reticulocytes. Parasitol. Res..

[55] Flygare J, Estrada VR, Shin C, Gupta S, Lodish HF (2011). HIF1 synergizes with glucocorticoids to promote BFU-E progenitor self-renewal. Blood.

[56] Bertini R, Bianchi M, Ghezzi P (1988). Adrenalectomy sensitizes mice to the lethal effects of interleukin 1 and tumor necrosis factor. J. Exp. Med..

[57] Ratman D (2013). How glucocorticoid receptors modulate the activity of other transcription factors: A scope beyond tethering. Mol. Cell. Endocrinol..

[58] Webster JI, Sternberg EM (2004). Role of the hypothalamic-pituitary-adrenal axis, glucocorticoids and glucocorticoid receptors in toxic sequelae of exposure to bacterial and viral products. J. Endocrinol..

[59] Hahner S (2010). Epidemiology of adrenal crisis in chronic adrenal insufficiency: the need for new prevention strategies. Eur. J. Endocrinol..

[60] Kotelevtsev Y (1997). 11Beta-hydroxysteroid dehydrogenase type 1 knockout mice show attenuated glucocorticoid-inducible responses and resist hyperglycemia on obesity or stress. Proc. Natl. Acad. Sci. USA.

