# Supplementary material for: 11β-hydroxysteroid dehydrogenase type 1 has no effect on survival during experimental malaria but affects parasitemia in a parasite strain-specific manner
Source: Sci Rep. 2017 Oct 23;7:13835. doi: 10.1038/s41598-017-14288-x (PMC5653823; doi:10.1038/s41598-017-14288-x)
Supplement: Supplementary file 1 — Supplementary information [file 41598_2017_14288_MOESM1_ESM.pdf]

11β-hydroxysteroid dehydrogenase type 1 has no effect on survival during experimental malaria but affects parasitemia in a parasite strain-specific manner

L. Vandermosten[1], C. De Geest[1], S. Knoops[1], G. Thijs[1], K.E. Chapman[2], K. De Bosscher[3], G Opdenakker[1], P.E. Van den Steen[1*]

[1] Laboratory of Immunobiology, Department of Microbiology and Immunology, Rega Institute for Medical Research, KU Leuven – University of Leuven, Leuven, Belgium

[2] University/BHF Centre for Cardiovascular Science, The Queen's Medical Research Institute, University of Edinburgh, Edinburgh, United Kingdom

[3] Receptor Research Laboratories, Nuclear Receptor Lab, VIB-UGent Center for Medical Biotechnology, Gent, Belgium

* Corresponding author: philippe.vandensteen@kuleuven.be

Supplementary information

[Supplementary Table S1.xls]

Supplementary Table S1. *Hsd11b1*$^{Del/Del}$ and WT littermates are highly similar to the C57Bl/6 genetic background

A genetic background characterization was performed on a panel of 1449 SNPs in both WT and *Hsd11b1*$^{Del/Del}$ mice to determine the percentage of C57Bl/6J genetic background. The *Hsd11b1*$^{Del/Del}$ mice and WT mice were, respectively, 99.93 - 100% and 100% similar to the C57Bl/6JOla11B-HSD1 reference genome. The *11β-HSD11b1* gene is located on mouse chromosome 1.

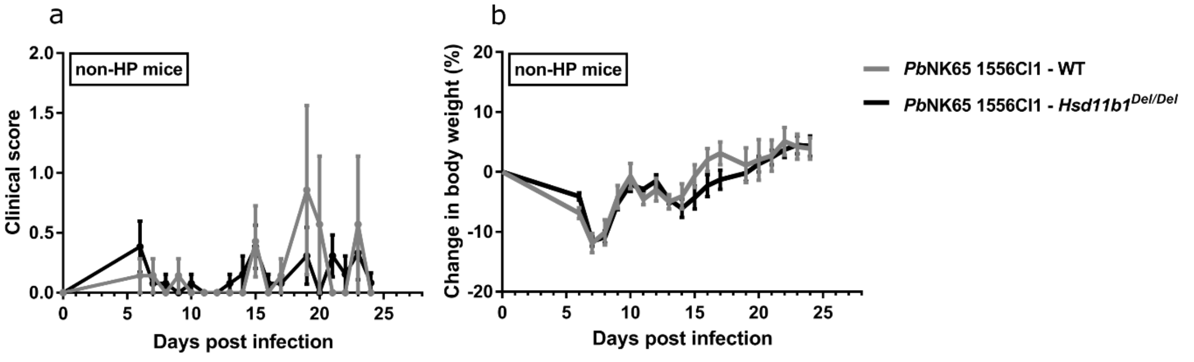

Supplementary Figure S1. 11β-HSD1 deficiency does not affect clinical score and body weight of PbNK65 1556Cl1–infected non-hyperparasitemic mice

Hsd11b1$^{Del/Del}$ and WT mice were infected with *Plasmodium berghei* NK65 (*Pb*NK65) clone 1556Cl1. The clinical score (a) and body weight change (b) of non-hyperparasitemic (non-HP) mice is shown. Data are means ± SEM and were analysed by Mann-Whitney U-test. No significant differences were found. Data from 2 separate experiments. n = 7 (4F and 3M) for WT and n = 13 (7F and 6M) for *Hsd11b1*$^{Del/Del}$.

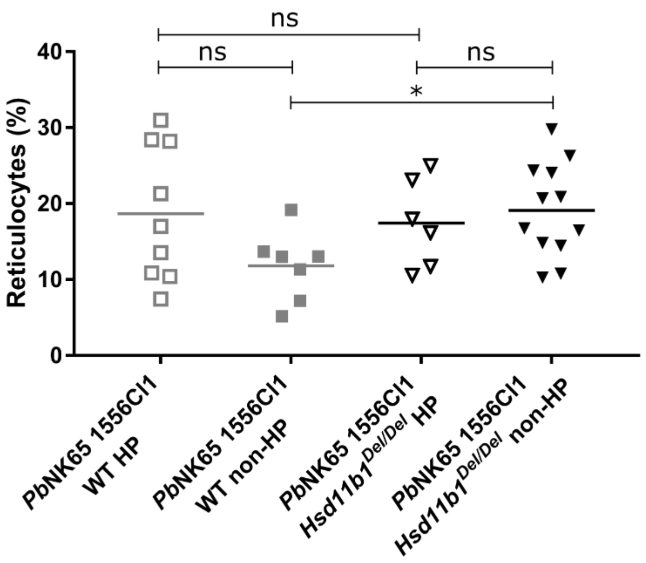

Supplementary Figure S2. Reticulocyte presence in HP and non-HP WT and *Hsd11b1*$^{Del/Del}$ mice infected with *Pb*NK65 1556Cl1

Hsd11b1$^{Del/Del}$ and WT mice were infected with *Plasmodium berghei* NK65 (*Pb*NK65) clone 1556Cl1. The percentage of reticulocytes amongst the RBCs was analysed by microscopical examination of blood smears at 17 days post-infection. Each dot represents the result from an individual mouse. Horizontal lines in between data points represent group medians and analysis was by Mann-Whitney U-test. Data from 2 separate experiments. n = 9 (3F and 6M) for HP WT, n = 7 (4F and 3M) for non-HP WT, n = 6 (2F and 4M) for HP *Hsd11b1*$^{Del/Del}$ and n = 12 (7F and 5M) for non-HP *Hsd11b1*$^{Del/Del}$. HP, hyperparasitemic; non-HP, non-hyperparasitemic; ns, not significant.

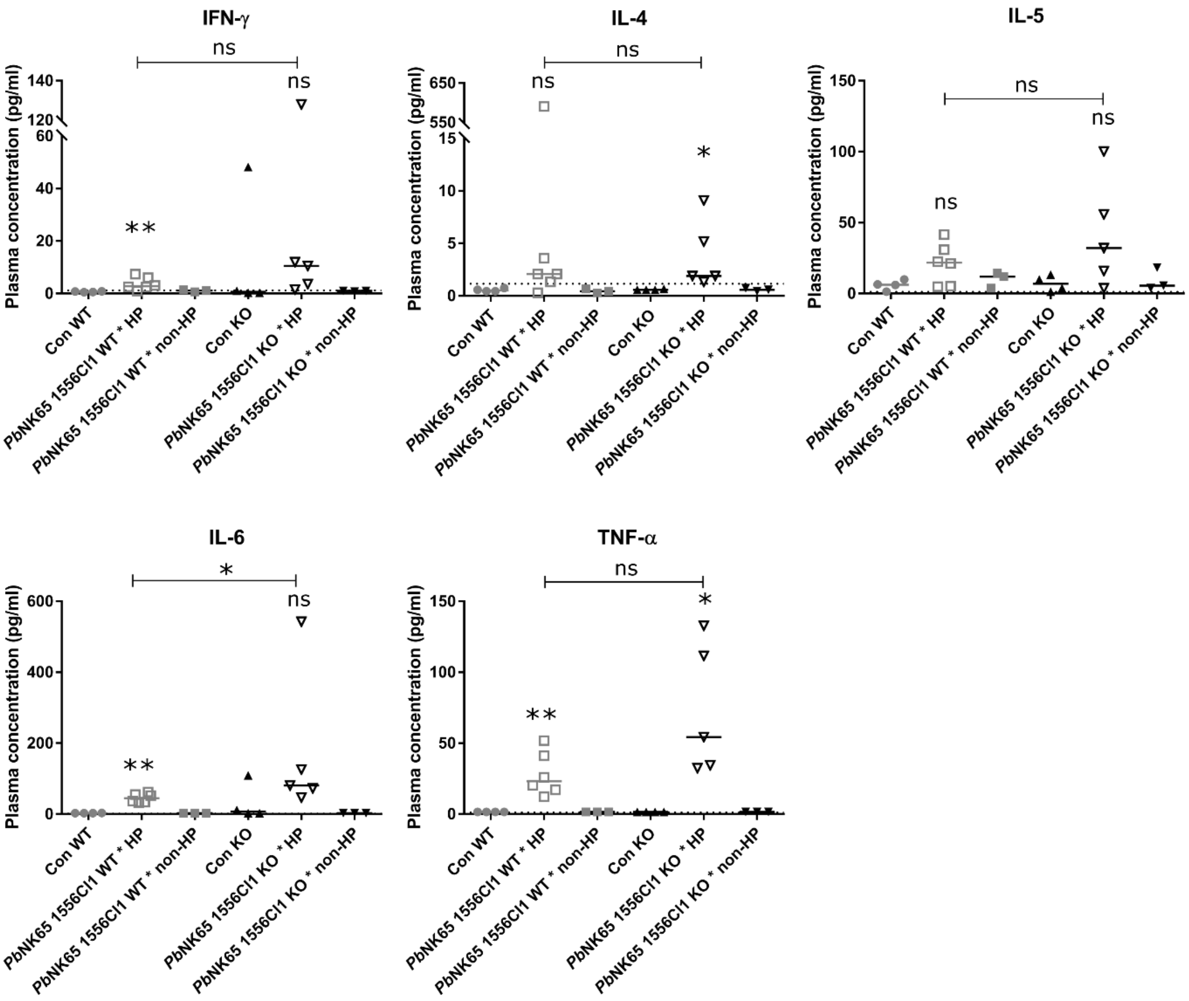

<u>Supplementary Figure S3. Effect of 11β-HSD1 on plasma levels of IFN-γ, IL-4, IL-5, IL-6 and TNF-α in *Pb*NK65 1556Cl1-infected mice</u>

Hsd11b1$^{Del/Del}$ and WT mice were infected with *Plasmodium berghei* NK65 (*Pb*NK65) clone 1556Cl1. Hyperparasitemic (HP) mice were dissected at 21-24 days post-infection (p.i.), non-hyperparasitemic (non-HP) mice at 28 days p.i.. Plasma levels of IFN-γ, IL-4, IL-5, IL-6 and TNF-α were determined and the dotted line indicates the limit of detection. Samples with a measurement below the detection limit, were given an arbitrary value of half of the detection limit. Each dot represents the result from an individual mouse. Horizontal lines in between data points represent group medians and analysis was by Mann-Whitney U-test. Asterisks above individual data sets indicate statistical differences compared to the uninfected control group. Data from 2 separate experiments. WT: n = 6 (3F and 3M) for HP; n = 3 (2F and 1M) for non-HP; *Hsd11b1*$^{Del/Del}$: n = 5 (2F and 3M) for HP; n = 3 (1F and 2M) for non-HP. ns, not significant.
